# Minimally invasive direct coronary artery bypass grafting with an improved rib spreader and a new-shaped cardiac stabilizer: results of 200 consecutive cases in a single institution

**DOI:** 10.1186/s12872-016-0216-4

**Published:** 2016-02-17

**Authors:** Yunpeng Ling, Liming Bao, Wei Yang, Yu Chen, Qing Gao

**Affiliations:** Department of Cardiac Surgery, Peking University Third Hospital, Beijing, China; Department of Cardiac Surgery, Aero Space Center Hospital, Beijing, China; Department of Cardiac Surgery, Peking University People’s Hospital, Beijing, China

**Keywords:** Minimally invasive direct coronary artery bypass (MIDCAB), Beating heart, Off-pump coronary artery surgery, Rib spreader, Cardiac stabilizer

## Abstract

**Background:**

Performing minimally invasive direct coronary artery bypass (MIDCAB) grafting via small chest incisions on a beating heart is challenging. We report our experiences of MIDCAB with the utilization of both an improved rib spreader to harvest the left internal mammary artery (LIMA) and a new-shaped cardiac stabilizer to facilitate LIMA-left anterior descending (LAD) coronary anastomosis.

**Methods:**

Between May 2012 and June 2104, a total of 200 patients who were consecutively operated on in this period were enrolled in this study. Data reported included demographic information, preoperative clinical and cardiac status, LIMA harvest time, postoperative in-hospital outcomes, and 30-day mortality.

**Results:**

The average LIMA harvest time was 43 min. The mean age was 62.59 ± 10.19 years, and 45 of the 200 were females. The 30-day mortality was 0.5 % (one patient) due to perioperative myocardial infarction. Duration of mechanical ventilation and length of stay in intensive care unit was 9.27 ± 7.65 and 24.27 ± 17.85 h, respectively. The unit of packed RBC transfusion was 0.79 ± 1.58. Postoperative atrial fibrillation was observed in 14 (7 %) patients. There was no postoperative stroke, renal failure, or incision complication.

**Conclusion:**

Performing MIDCAB with the improved retractor and stabilizer utilized in this study showed favorable outcomes in terms of harvesting the LIMA, postoperative morbidities, and 30-day mortality.

## Background

Minimally invasive direct coronary artery bypass (MIDCAB) grafting attempts to achieve adequate coronary artery revascularization in a less invasive manner than conventional coronary artery bypass grafting (CABG). Unlike conventional revascularization techniques, which are highly invasive due to the use of a large incision (sternotomy) and cardiopulmonary bypass (CPB), MIDCAB limits invasiveness by employing a small incision (thoracotomy) and by operating on the beating heart to avoid the need for CPB [1]. By limiting invasiveness in these ways, MIDCAB can reduce the risk of complications such as infection and stroke [2]. In comparison to conventional CABG and off-pump CABG (via a sternotomy), MIDCAB can improve early post-operative quality of life [3] and recovery time [4], respectively.

While MIDCAB can lead to favorable outcomes, performing coronary anastomosis via a small chest incision on a beating heart can be challenging [5]. This makes it difficult to accomplish two key aspects of the MIDCAB procedure. The first pertains to obtaining an adequate length of left internal mammary artery (LIMA) [6] and the second is in regard to adequately stabilizing the wall of the beating heart, without adversely affecting hemodynamics or injuring the myocardium [7]. To overcome these difficulties, a variety of retractors and cardiac stabilizers have been utilized, but there is interest in improving their design [8].

Recently, we started utilizing a Fehling retractor (Fehling, Germany), a rib spreader, to harvest the LIMA under direct vision. Compared to existing retractors, this improved retractor allows for an expanded field of vision and enhances LIMA exposure [9]. Additionally, we started using a newly developed stabilizer during MIDCAB. Compared to existing stabilizers, this stabilizer has a better shape to facilitate anastomosing the deeply located left anterior descending (LAD) coronary artery, and can be applied with light pressure, thereby reducing the risk of adverse hemodynamic effects and myocardial injury. In theory, these new and improved devices might lead to improved patient outcomes during MIDCAB, but empirical data are needed to fully assess this issue. Accordingly, the present study reported outcomes in patients who underwent MIDCAB using both the Fehling retractor and the improved cardiac stabilizer.

## Methods

This descriptive, non-experimental study included a total of 200 consecutive patients, who were scheduled to undergo a MIDCAB operation at our institution between May 2012 and June 2014. All patients were treated by a single surgeon at the Department of Cardiac Surgery of Peking University People’s Hospital. The study was approved by the institutional ethics committee of the Department of Cardiac Surgery of Peking University People’s Hospital, and all study patients provided written informed consent prior to surgery.

### Surgical procedures and technique

**Fig. 1:**
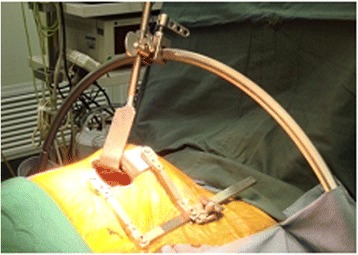
Suspensory internal mammary artery retraction system

Double-cavity tracheal cannulas were used along with general anesthesia. Patients were placed in the supine position, with the left chest raised by 30°. A 5–6 cm surgical incision was then made in the area of the fourth or fifth rib, and the thoracic cavity was entered. To enable LIMA harvesting, a specialized suspensory internal mammary artery retraction system was utilized (Fig. 1). This retractor offers advantages compared to other retractors in that it can integrally raise the left chest wall, thereby avoiding excessive traction of local ribs while simultaneously providing a good operative field to facilitate obtaining adequate LIMA. LIMA was harvested from the upper segment to the lower segment, and LIMA branches were clipped using a pen titanium clamp.

**Fig. 2:**
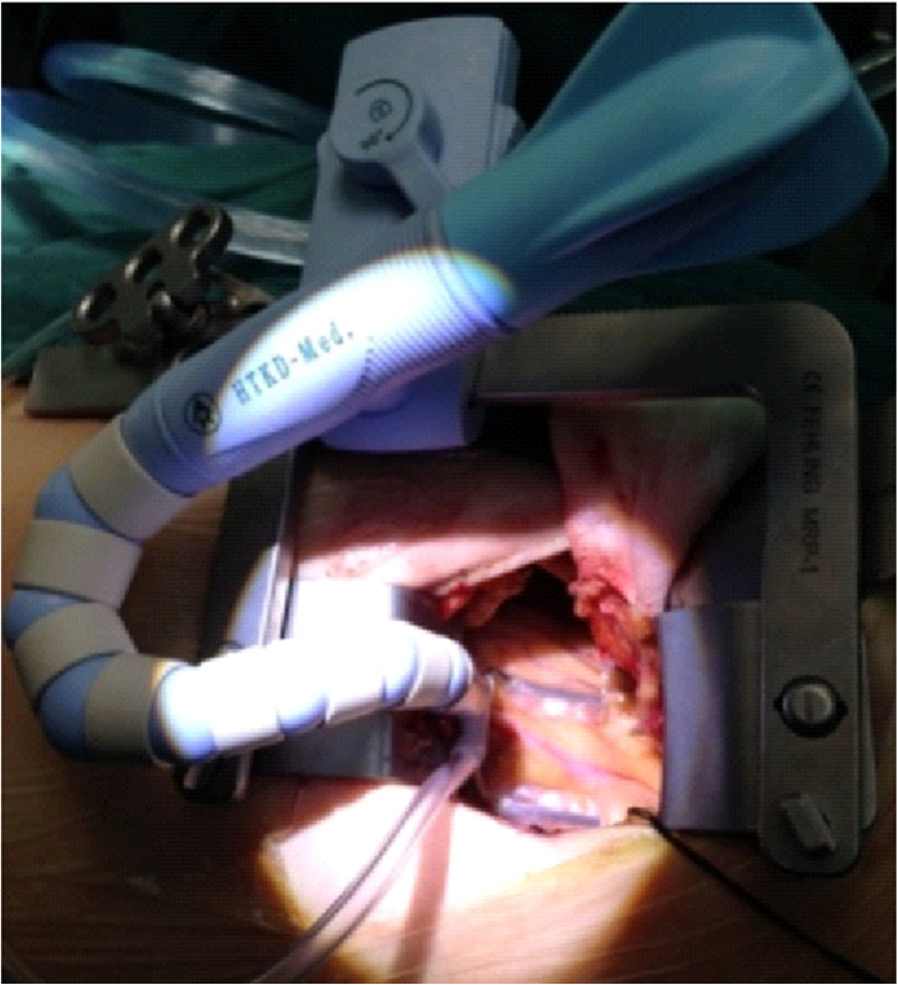
L-shaped suction stabilizer foot

**Fig. 3:**
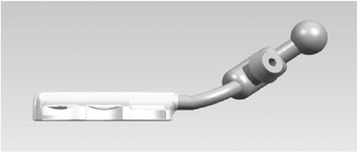
Schematic diagram of cardiac stabilizer

Beating heart LIMA-LAD anastomosis was facilitated by a heart stabilizer specially designed for MIDCAB procedures (HK, Figs. 2 and 3). This stabilizer is unique in that its presser foot has an “L”-shape, which is particularly advantageous for procedures performed within a deep space, such as MIDCAB. A second advantage of this stabilizer is that it can be placed on a rib retractor without an external fixator. Because of these two advantages, the stabilizer’s presser foot only needs to be in light contact with the epicardium to achieve stability through negative pressure suction. This light contact limits squeezing pressure on the heart, and thereby reduces the risk of circulatory instability. Additionally, the stabilizer has a reduced number of adsorption holes and a reduced adsorption area on the sucker, both of which help to limit the myocardial area that could potentially be damaged by the stabilizer. After incising the coronary artery, routine coronary artery bypass grafting was performed, and 8-0 prolene lines were utilized to perform a continuous suture. All of the 200 study subjects underwent LIMA-LAD single bypass during the MIDCAB.

### Data collection

Prior to surgery, subjects’ height and weight were measured and questionnaires were administered to collect patient’s age, gender, history of medical conditions, and history of cardiovascular procedures. Pre-operative measurements were made of each patient’s ejection fraction and angiographic status and stored in a hospital database for subsequent access. Study personnel also collected data on procedure duration and other in-hospital outcomes and complications. After the patients were discharged from the hospital, they were followed up as outpatients to obtain the information regarding 30-day mortality. Data were available on all 200 patients. Patient data were examined and presented by descriptive statistics. Setting the Type I error rate at 0.05 and the drop-out rate at 10 %, a power analysis [10] showed that to detect a proportion of 0.07, a sample size of 194 patients would be sufficient to achieve a statistical power of 80 % at a 5 % significance level. This study included a total of 200 patients, which satisfied the required sample based on the post hoc analysis.

## Results

The average LIMA harvest time was 43 min. The preoperative characteristics of the patients are displayed in Table 1. The patients included 138 individuals that received MIDCAB alone and 62 individuals that underwent hybrid procedure (MIDCAB for LAD, plus percutaneous coronary intervention (PCI) for other blood vessels). The mean age was 62.59 ± 10.19 years, and 45 of the 200 were females.

**Table 1:** Preoperative patient characteristics (*N* = 200)

	MIDCAB	Hybrid	Total
	(*n* = 138)	(*n* = 62)	(*N* = 200)
Age, year	63.65 ± 10.48	60.24 ± 9.18	62.59 ± 10.19
Sex, N (%) of female	35 (25.4 %)	10 (16.1 %)	45 (22.5 %)
Height, cm	167.08 ± 7.4	168.3 ± 6.32	167.45 ± 7.1
Weight, kg	70.69 ± 10.53	71.73 ± 9.52	71.01 ± 10.21
Hypertension, N (%)	80 (58.0 %)	26 (41.9 %)	106 (53.0 %)
Diabetes mellitus, N (%)	40 (29.0 %)	27 (43.5 %)	67 (33.5 %)
Smoking, N (%)	63 (45.7 %)	33 (53.2 %)	96 (48.0 %)
Hypercholesterolemia, N (%)	20 (14.5 %)	9 (14.5 %)	29 (14.5 %)
Old MI, N (%)	40 (29.0 %)	17 (27.4 %)	57 (28.5 %)
PCI history, N (%)	17 (12.3 %)	0 (0.0 %)	17 (8.5 %)
Renal insufficiency, N (%)	4 (2.9 %)	0 (0.0 %)	4 (2.0 %)
NYHA grade 1-2, N (%)	120 (87.0 %)	55 (88.7 %)	175 (87.5 %)
NYHA grade 3, N (%)	18 (13.0 %)	7 (11.3 %)	25 (12.5 %)
LVEF, N (%)			
> 55 %	114 (82.6 %)	52 (83.9 %)	166 (83.0 %)
46–55 %	16 (11.6 %)	3 (4.8 %)	19 (9.5 %)
36–45 %	7 (5.1 %)	6 (9.7 %)	13 (6.5 %)
≤ 35 %	1 (0.7 %)	1 (1.6 %)	2 (1.0 %)
LVEDd, mm	50.16 ± 5.96	50.52 ± 5.76	50.27 ± 5.89
Single-vessel disease	89 (64.5 %)	0 (0.0 %)	89 (44.5 %)
Left main or multi-vessel disease	49 (35.5 %)	62 (100.0 %)	111 (55.5 %)

LVEF, left ventricular ejection fractions; LVEDd, left ventricular end diastolic diameter; MI, myocardial infarction; NYHA, New York Heart Association; PCI, Percutaneous coronary intervention

Table 2 shows the in-hospital outcomes and 30-day mortality. One (0.5 %) patient died within 30 days due to perioperative myocardial infarction (MI). Duration of mechanical ventilation was 9.27 ± 7.65 h and length of stay in intensive care unit was 24.27 ± 17.85 h. The unit of packed RBC transfusion was 0.79 ± 1.58. Postoperative atrial fibrillation (PAF) was observed in 14 (7 %) patients. No patient had experienced stroke, renal failure, or incision complication.

**Table 2:** In-hospital clinical outcomes and 30-day mortality (*N* = 200)

	MIDCAB	Hybrid	Total
	(*n* = 138)	(*n* = 62)	(*N* = 200)
30-day mortality, N (%)	1 (0.7 %)	0 (0.0 %)	1 (0.5 %)
Perioperative MI, N (%)	1 (0.7 %)	0 (0.0 %)	1 (0.5 %)
Duration of mechanical ventilation, hour	9.93 ± 8.65	7.79 ± 4.43	9.27 ± 7.65
LOS in ICU, hour	24.17 ± 17.83	24.48 ± 18.03	24.27 ± 17.85
PRBC, units	0.86 ± 1.63	0.61 ± 1.47	0.79 ± 1.58
PAF, N (%)	10 (7.2 %)	4 (6.5 %)	14 (7.0 %)
Stroke, N (%)	0 (0.0 %)	0 (0.0 %)	0 (0.0 %)
Renal failure, N (%)	0 (0.0 %)	0 (0.0 %)	0 (0.0 %)
Incision complications, N (%)	0 (0.0 %)	0 (0.0 %)	0 (0.0 %)

ICU, intensive care unit; LOS, length of stay; MI, myocardial infarction; PAF, postoperative atrial fibrillation; PRBC, packed red blood cell

## Discussion

In this study of the MIDCAB procedure using an improved retractor and stabilizer, we found the following in-hospital outcomes: an average LIMA harvest time of 43 min, a mean duration of mechanical ventilation of 9 h, and a mean ICU stay of 24 h. PAF occurred in only 7 % of the patients, and there was no postoperative stroke, renal failure, or incision complications. The 30-day mortality rate was 0.5 %. Together, these data suggest that performing MIDCAB with the improved retractor and stabilizer used in this study can lead to favorable outcomes.

The MIDCAB procedure was introduced into the surgical literature in 1995 [11]. Subsequently, MIDCAB was adopted by various universities and hospitals in Europe and America [12]. Reports indicate that MIDCAB not only has comparable patency rates with off-pump CABG (via sternotomy) [13] and conventional CABG [14] but leads to favorable outcomes such as shorter hospitalization [4], faster recovery [4], and less need for blood transfusion [15]. Initially, MIDCAB procedures were performed using chest wall retraction under direct vision. However, retraction of the chest wall could lead to postoperative pain and obtaining LIMA under direct vision could be technically challenging. Consequently, some centers started to perform MIDCAB using robotic surgery, yet the robotically assisted approach has a significant learning curve and major costs [11]. As such, MIDCAB using chest wall retraction under direct vision remains a reasonable, affordable approach for developing nations [16, 17] such as China, but concerns remain about pain due to the retraction and about hemodynamic instability and myocardial injury due to the use of cardiac stabilizers.

To address concerns about retraction-associated pain, hemodynamic instability, and myocardial injury, we performed MIDCAB operations during the past two years using an improved retractor and a new-shaped cardiac stabilizer. The retractor is a suspensory internal mammary artery retraction system that facilitates obtaining LIMA under direct vision. In particular, this retractor raises the anterior chest wall integrally, which allows the surgeon to work within a good operative space without having to retract the chest wall excessively. This allows for adequate LIMA harvesting while limiting pain due to retraction. The newly developed stabilizer has an L-shaped presser foot, the shape of which is advantageous for operating within the deep but narrow opening in which MIDCAB procedures take place; furthermore, the stabilizer can be placed on a rib spreader without an external fixator. As such, the stabilizer need only exert light pressure on the epicardium in order to achieve adequate coronary stabilization through negative pressure suction. In this way, the stabilizer reduces the risk of adverse hemodynamic events. Moreover, the stabilizer has a relatively small number of adsorption holes covering a relatively small area on the sucker, and therefore, reduces the area of myocardium that is at risk of damage from stabilizer-induced contact.

Data from our study suggest that MIDCAB procedures performed with the improved retractor and stabilizer had favorable outcomes. The average time to obtain LIMA was 43 min, which is shorter than in some studies using robotic devices. Fujita et al. successfully performed MIDCAB surgery with robotic LIMA harvesting in 30/33 patients; their average harvest time was 68 min [18]. In another report of robotic endoscopic LIMA harvesting in 100 cases, the reported median harvest time was 48 min; however, there was a significant learning curve so that the harvest time decreased with experience, from 140 min in the first 10 cases to 40 min in the last 10 cases [19]. The harvest time of 43 min in the current study using an improved retractor and stabilizer compares favorably with these two reports using robotic techniques.

Internal mammary artery injuries were not found in any of the patients in the current study. In Fujita’s study, 3/33 patients (9 %) had bleeding from the LIMA requiring conversion to a median sternotomy [18]. Additionally, there were 4 cases of LIMA injury in Oehlinger’s series, 3 (6 %) during the first half of their experience and 1 (2 %) during the second half. Additionally, one patient required median sternotomy because of LIMA injury during robotic harvesting [19]. Thus, the technique in the current study provided better results in terms of arterial injuries compared with these prior studies using robotic techniques.

Mechanical ventilation is another parameter of concern. A retrospective analysis of 217 patients who underwent MIDCAB in Germany was performed with a focus on fast-track recovery [20]. In that study, extubation was performed immediately after surgery in 182 (83.9 %) patients, only 8 of whom required re-intubation within one hour of arrival in the ICU. Of the remaining 35 patients, 31 required ventilation for <24 h, and 4 patients required >72 h of ventilation [20]. Another group in Germany compared their results for patients undergoing surgery with a full sternotomy (OPCAB, *n* = 44) and those undergoing MIDCAB procedures (n = 58); they generally found that MIDCAB was more challenging and had worse results. For example, although there was no perioperative mortality in either group, time on the ventilator was longer in the MIDCAB compared with the OPCAB group (29 ± 109 h vs 10 ± 6 h, NS) [21]. The time in the ICU was 57 ± 129 h vs 32 ± 14 h for the OPCAB and MIDCAB groups, respectively [21]. Bisbos et al. reported their experience with MIDCAB in 91 patients in Greece; the mean ICU stay was 29 ± 4 h [22]. In the current study, the duration of mechanical ventilation was 9.93 ± 8.65 h, and the length of ICU stay was 24.17 ± 17.83 h, again comparing favorably with prior studies.

In-hospital complications with MIDCAB procedures may include PAF, stroke, renal failure, and incision complications. McGinn et al. reported on the results of 450 minimally invasive coronary artery bypass surgeries at two centers in the US and found the following complications: stroke, *n* = 2 (0.4 %); new-onset atrial fibrillation, *n* = 111 (24.4 %); new-onset renal failure, *n* = 12 (2.9 %); and wound infection, *n* = 4 (0.9 %) [23]. In the current study, the rates of these complications were less: only 7 % of patients developed atrial fibrillation, and no patients developed stroke, renal failure, or incision complications.

Additionally, the 30-day mortality rate was less than 1 % in the current study. In a report of the experience with MIDCAP cases from a single center in the Czech Republic, there was a 30-day mortality rate of 1.3% (2/149 patients) [24]. Similarly, an early (<30 days) mortality rate of 1.3 % (51/4081 patients) was reported in a meta-analysis of 17 studies utilizing MIDCAB grafting [25]. The 30-day mortality rate in the current study compares favorably with these results.

There are several limitations of this study. First, it is observational in nature, with the inherent bias involved in such studies. Also, there was no direct comparison group, so that all comparisons were with the published literature. Since the patients in these prior publications may not be similar populations, firm conclusions cannot be made although the findings in the current study seemed to compare favorably with historical results. Significantly, no patency rates, long-term follow-up or long-term outcomes were available from the current study. Therefore, further studies are needed to better define the full range of outcomes with the new techniques described in the current study.

## Conclusions

Taken together, the two devices have the following clinical benefits. The suspensory internal mammary artery retraction system utilized in the current study shortens the LIMA harvest time and minimizes the risk of damaging the internal mammary arteries. In addition, the retractor allows for obtaining the LIMA under direct vision, which facilitates harvesting an adequate length of LIMA via a small surgical incision in the left side of the chest. The newly developed cardiac stabilizer is advantageous in regard to maintaining intraoperative circulatory stability and reducing the risk of injuring the myocardium. Furthermore, this study suggests that performing MIDCAB with the improved retractor and stabilizer can lead to favorable outcomes in terms of postoperative morbidity and 30-day mortality.

## Abbreviations

CABG: Coronary artery bypass grafting
CPB: Cardiopulmonary bypass
LAD: LIMA-left anterior descending
LIMA: Left internal mammary artery
MI: Myocardial infarction
MIDCAB: Minimally invasive direct coronary artery bypass
PAF: Postoperative atrial fibrillation
PCI: Percutaneous coronary intervention
